# Rapid Diagnosis of Bloodstream Infections with PCR Followed by Mass Spectrometry

**DOI:** 10.1371/journal.pone.0062108

**Published:** 2013-04-23

**Authors:** Elena Jordana-Lluch, Heather E. Carolan, Montserrat Giménez, Rangarajan Sampath, David J. Ecker, M. Dolores Quesada, Josep M. Mòdol, Fernando Arméstar, Lawrence B. Blyn, Lendell L. Cummins, Vicente Ausina, Elisa Martró

**Affiliations:** 1 Microbiology Department, Fundació Institut d’Investigació en Ciències de la Salut Germans Trias i Pujol, Hospital Universitari Germans Trias i Pujol, Universitat Autònoma de Barcelona, Badalona, Spain; 2 Ibis Biosciences, an Abbott company, Carlsbad, California, United States of America; 3 Emergency Room, Hospital Universitari Germans Trias i Pujol, Badalona, Spain; 4 Intensive Care Unit, Hospital Universitari Germans Trias i Pujol, Badalona, Spain; 5 CIBER Enfermedades Respiratorias (CIBERES), Bunyola, Spain; 6 CIBER Epidemiología y Salud Pública (CIBERESP), Barcelona, Spain; Charité-University Medicine Berlin, Germany

## Abstract

Achieving a rapid microbiological diagnosis is crucial for decreasing morbidity and mortality of patients with a bloodstream infection, as it leads to the administration of an appropriate empiric antimicrobial therapy. Molecular methods may offer a rapid alternative to conventional microbiological diagnosis involving blood culture. In this study, the performance of a new technology that uses broad-spectrum PCR coupled with mass spectrometry (PCR/ESI-MS) was evaluated for the detection of microorganisms directly from whole blood. A total of 247 whole blood samples and paired blood cultures were prospectively obtained from 175 patients with a suspicion of sepsis. Both sample types were analyzed using the PCR/ESI-MS technology, and the results were compared with those obtained by conventional identification methods. The overall agreement between conventional methods and PCR/ESI-MS performed in blood culture aliquots was 94.2% with 96.8% sensitivity and 98.5% specificity for the molecular method. When comparing conventional methods with PCR/ESI-MS performed in whole blood specimens, the overall agreement was 77.1% with 50% sensitivity and 93.8% specificity for the molecular method. Interestingly, the PCR/ESI-MS technology led to the additional identification of 13 pathogens that were not found by conventional methods. Using the PCR/ESI-MS technology the microbiological diagnosis of bloodstream infections could be anticipated in about half of the patients in our setting, including a small but significant proportion of patients newly diagnosed. Thus, this promising technology could be very useful for the rapid diagnosis of sepsis in combination with traditional methods.

## Introduction

The administration of an empiric antibiotic therapy within the first hour of recognition of clinical symptoms of bloodstream infections is strongly recommended [1]. However, a rapid microbiological diagnosis is of paramount importance for the best outcome of the patient, since it allows for the administration of an appropriate empiric treatment on the basis of the clinician’s knowledge of the antimicrobial susceptibility status of each bacterial species that is prevalent in that area. Once the identification is achieved, the initial therapy can be changed, if necessary, to assure an adequate antibiotic activity against the etiologic agent, or its spectrum reduced to prevent antimicrobial resistance development [2]. While susceptibility testing results are usually delayed 42–72 h, every hour gained to initiate proper antimicrobial therapy significantly increases the probability of patient survival [3].

The reference method used for the detection of pathogens in blood of septic patients is the blood culture (BC) followed by conventional identification methods. This methodology, while being necessary for the assessment of antimicrobial susceptibility, implies a delay of up to 48–72 h in the identification of the etiologic agent. Furthermore, despite being the reference diagnostic method, the BC has several limitations regarding sensitivity, especially in the case of previous antimicrobial therapy, and fastidious, slow-growing or uncultivable pathogens, often leading to a low diagnostic yield [4].

In order to speed the diagnostic process, it would be desirable to detect and identify the microorganisms directly from the patient’s blood avoiding culture. Currently, molecular methods offer a rapid and reliable alternative to conventional culture, reducing the time to detection and increasing the sensitivity in the identification of certain microorganisms. As N. Mancini *et al.* recently reviewed [4], several assays are commercially available for the detection and identification of microorganisms related to bloodstream infections directly from whole blood (WB). Among them, only SeptiFast (Roche, Mannheim, Germany) has been evaluated in several studies in the hospital setting leading to heterogeneous results [5]–[10]. Besides, this assay is limited to the 25 pathogens most commonly involved in sepsis; however, this syndrome can be caused by a broad range of pathogens and, thus, the diagnostic value is limited to the detection of the microorganisms included in the assay.

Recently, a new and promising technology has been described that uses a broad-spectrum PCR coupled with electrospray ionization mass spectrometry (PCR/ESI-MS; Ibis Biosciences, an Abbott company, Carlsbad, CA, USA) to potentially identify any microorganism present in a clinical specimen [11]–[13]. Using mass spectrometry, the mass of each PCR amplicon is determined and the nucleotide base composition is unambiguously calculated and compared to a database, achieving the identification. This technology has shown promising results in the accurate detection of microorganisms directly from clinical specimens, including the detection of respiratory pathogens [14]–[16]. The goal of the present study was to test in a clinical setting the performance of this new technology for the identification of sepsis-related pathogens directly from WB. However, given the low concentration of microorganisms in this specimen type, a first evaluation was carried out on BC to confirm the ability of the PCR/ESI-MS technology to identify a variety of sepsis-related pathogens, and then its performance was assessed on WB.

## Materials and Methods

### Ethics Statement

Written informed consent was obtained from all patients or their guardians. This study was approved by the Clinical Research Ethics Committee at our institution (“Comité Ético de Investigación Clínica”, CEIC).

### Patients and Specimens

This was an observational study where a total of 175 patients with a suspicion of sepsis according to the American College of Chest Physicians/Society of Critical Care Medicine (ACCP/SCCM) criteria [17] were prospectively included between April 2010 and May 2011 (median age 60 years, range 1–94; 70 male and 102 female).

For each patient, a WB specimen was collected in an EDTA tube under aseptic conditions at the onset of fever or other clinical signs of sepsis, when the BC was inoculated for routine microbiological testing; for 42 patients, serial specimens were included, either from the same or different sepsis episodes, adding to a total of 247 WB specimens tested. For a subset of 206 WB specimens, an aliquot of the paired BC bottle/s was also obtained after incubation in order to compare the performance of the molecular method in the two specimen types. WB specimens were classified depending on their paired BC result as WB with a paired positive BC (n = 75) or WB with a paired negative BC (n = 172). WB specimens were frozen at −20°C for up to nine months until DNA extraction. Long term stability of WB samples under these storage conditions had previously been demonstrated by the manufacturer on spiked samples.

### Conventional Microbiological Methods

For each adult patient, a set of two BC, including two aerobic and one anaerobic BC bottles, were inoculated with up to 10 mL of blood whereas the pediatric BC were filled with up to 2–5 mL depending on the child’s age. The BC bottles were incubated in the Bactec 9240 BC system (Becton Dickinson, Franklin Lakes, NJ, USA) for up to 5 days before being called negative. The identification and susceptibility testing of the microorganism/s present in the positive BC bottles was achieved using the Vitek-2 Compact system (BioMérieux, Marseille-L’Étoile, France) after performing a Gram stain and a concentration protocol [18], [19]. Conventional cultures were also performed following standard microbiological methods for identification and antibiotic susceptibility testing (disc diffusion and minimum inhibitory concentration methods as required).

### DNA Extraction

For each patient DNA was extracted from 1.250 mL of WB and from 1.1 mL of the BC aliquot, according to the manufacturer’s instructions. After a mechanical lysis step with the Precellys instrument (Bertin Technologies, Montigny-le-Bretonneux, France), the DNA was extracted using the magnetic-bead based extractor KingFisher Flex (ThermoScientific, Waltham, MA, USA) following the manufacturer’s protocols. The eluted DNA (200 µL) was blind-coded regarding both the patient’s identity and the BC result and frozen at −80°C for further analysis at Ibis Biosciences (Carlsbad, CA, USA).

### DNA Amplification and Analysis

Two investigational assays were used to amplify DNA extracts from WB and BC specimens, respectively, according to the manufacturer’s protocols. The assays were designed to detect a broad range of bacteria and *Candida* spp. as well as four antibiotic resistance genes: *mecA* (resistance to methicillin), *vanA* and *vanB* (resistance to vancomycin) and *kpc* (resistance to carbapenems) in a 96-well plate. After DNA amplification in a Mastercycler® ep S Thermal Cycler (Eppendorf, Eppendorf AG. Hamburg, Germany), according to the manufacturer’s protocol, the plates were transferred to the ESI-MS instrument (Ibis Biosciences). The PCR products were analyzed using electrospray ionization time of flight mass spectrometry (ESI-TOF) to determine the mass of each amplicon strand. Using a built-in software analysis package, the base count of each amplicon was deduced from the measured masses and compared with the reference database. The combination of the results obtained from each primer pair was used to identify the microorganism/s present in the sample. Furthermore, the genetic material was quantified by using an internal calibrant added at a known concentration to each PCR well. Identification can be obtained from clinical specimens in 6–8 hours including DNA extraction, PCR amplification and ESI-TOF analysis.

### Data Interpretation and Statistical Analysis

For each patient the results obtained with the PCR/ESI-MS technology on WB and its paired BC were compared with those obtained using conventional methods (BC was considered the gold standard). As discrepancies between both methods were found, to assess whether the microorganisms identified only by the PCR/ESI-MS corresponded to the true etiological agents of the sepsis episode and, thus, had clinical significance, the results were compared to a constructed “clinical infection criterion”; this new gold standard was based on clinical records review in order to identify the diagnosed focus of infection, as well as on the results of cultures from other specimens (i.e. microorganisms detected only by PCR/ESI-MS were considered true positives when the same microorganism had been isolated from a culture from another specimen type reflecting the focus of infection or supported by the nature of the underlying infection).

Since more than one microorganism could be detected per specimen, the methods were compared at two levels against the two aforementioned gold standards: 1) the Cohen’s Kappa coefficient of agreement was calculated (OpenEpi software) [20] between both methods considering all microorganisms identified (a direct comparison for each microorganism isolated by conventional methods vs. the same microorganism detected by the molecular method); and 2) positive and negative results by each method were computed in order to calculate the parameters of analytical performance of the molecular method (sensitivity, specificity, positive and negative predictive values), excluding specimens with polymicrobial detections, since they could not be properly classified (i.e. both methods agreed in some but not all microorganisms identified).

## Results

### 1. Microbial Identification from Blood Culture Specimens

The performance of the PCR/ESI-MS at identifying a variety of sepsis-related microorganisms was firstly assessed on BC aliquots in comparison with conventional methods.

#### Agreement between isolated/detected microorganisms

When the PCR/ESI-MS was compared to the conventional methods using the BC as the gold standard, the same microorganism was identified in 78 out of a total of 96 identifications by either or both methods, while 128 specimens were negative by both methods. Thus, the overall agreement at the microorganism level was 92.0% (κ = 0.830) (**Table 1A**). Polymicrobial infections involving two or three microorganisms were detected by either or both methods in 14 (18.7%) cases among the 75 specimens with a positive BC (**Table 2**). Six of the seven microorganisms detected by BC that were missed by PCR/ESI-MS corresponded to polymicrobial specimens. However, the PCR/ESI-MS technology detected an additional five microorganisms not detected by BC that were clinically significant (**Table 3**). Thus, when a clinical infection criterion was used as the gold standard, the agreement rose to 94.2% (κ = 0.879) (**Table 1A**).

**Table 1:** Agreement between microorganisms isolated by conventional microbiological methods and detected by the PCR/ESI-MS method according to the gold standard used and the specimen type.

		Blood culture gold standard			Clinical infection criterion		
		Conventional methods			Conventional methods		
		Positive	Negative	Total	Positive	Negative	Total
**A) PCR/ESI-MS in blood culture**	Positive	78	11	**89**	83	6	**89**
	Negative	7	128	**135**	7	128	**135**
	**Total**	**85**	**139**	**224**	**90**	**134**	**224**
**B) PCR/ESI-MS in whole blood**	Positive	37	25	**62**	50*	12	**62**
	Negative	48	152	**200**	48	152	**200**
	**Total**	**85**	**177**	**262**	**98**	**164**	**262**

**A)** Overall agreement, blood culture gold standard: [(78+128)/224] = 92.0%, and clinical infection criterion: [(83+128)/224] = 94.2%.

**B)** Overall agreement, blood culture gold standard: [(37+152)/262] = 72.1%, and clinical infection criterion: [(50+152)/262] = 77.1%.

* Two detections correspond to different specimens from the same patient and sepsis episode.

**Table 2:** Polymicrobial infections/detections by conventional methods or PCR/ESI-MS according to specimen type.

		PCR/ESI-MS	
Specimen	Conventional methods	Blood culture	Whole blood
1	*Klebsiella pneumoniae*	*Klebsiella pneumoniae*	*Klebsiella pneumoniae*
	*Enterococcus faecium*	*Enterococcus faecium*	*Klebsiella oxytoca*
2	*Enterococcus faecalis*	*Enterococcus faecalis*	Not detected
	*Pseudomonas aeruginosa*	Not detected	*Pseudomonas aeruginosa*
	*Klebsiella pneumoniae*	Not detected	Not detected
3	*Citrobacter koseri*	*Citrobacter* spp.	*Citrobacter koseri*
	*Hafnia alvei*	Not detected	Not detected
4	*Enterobacter cloacae*	*Enterobacter cloacae* complex	*Enterobacter intermedius*
	*Enterococcus faecalis*	Not detected	Not detected
	CoNS^1^	CoNS	Not detected
5	*Streptococcus parasanguinis*	*Streptococcus* spp.	*Streptococcus thermophylus*
	*Staphylococcus epidermidis*	CoNS	Not detected
6	*Escherichia coli*	*Escherichia coli*	Not detected
	*Staphylococcus aureus*	*Staphylococcus aureus*	Not detected
7	*Serratia marcescens*	*Serratia marcescens*	Not detected
	*Enterococcus faecium*	Not detected	Not detected
8	*Escherichia coli*	*Escherichia coli*	*Escherichia coli*
	*Staphylococcus epidermidis*	CoNS	*Staphylococcus epidermidis*
9	*Klebsiella pneumoniae*	*Klebsiella pneumoniae*	Not detected
	Not detected	*Citrobacter* spp.	Not detected
	Not detected	Not detected	*Staphylococcus warneri*
10	*Fusobacterium* spp.	*Fusobacterium* spp.	Not detected
	Not detected	*Clostridium* spp.	Not detected
11	*Staphylococcus epidermidis*	CoNS	Not detected
	Not detected	*Enterobacter cloacae* complex	Not detected
12	*Enterococcus faecalis*	*Enterococcus faecalis*	Not detected
	Not detected	*Enterobacter cloacae* complex	Not detected
13	*Bacteroides* spp.	*Bacteroides fragilis*	*Bacteroides fragilis*
	Not detected	*Clostridium* spp.	Not detected
14	*Serratia* spp.	*Serratia* spp.	Not detected
	Not detected	*Bacillus* spp.	Not detected
15	*Enterococcus faecium*	*Enterococcus faecium*	*Enterococcus faecium*
	Not detected	Not detected	*Candida albicans*
16	*Pseudomonas aeruginosa*	*Pseudomonas aeruginosa*	*Pseudomonas aeruginosa*
	Not detected	Not detected	*Corynebacterium* spp.
17	*Escherichia coli*	*Escherichia coli*	*Escherichia coli*
	Not detected	Not detected	*Enterobacter cloacae*

1 *Staphylococcus epidermidis* and other coagulase-negative species (CoNS).

**Table 3:** Pathogens with clinical significance isolated by conventional microbiological methods and detected by the PCR/ESI-MS method.

		N° of microorganisms detected					
		Blood culture specimens			Whole blood specimens		
Group	Microorganism	BConly	PCR/ESI-MSand BC	PCR/ESI-MSonly	BConly	PCR/ESI-MSand BC	PCR/ESI-MSonly
**Gramnegatives**	*Bacteroides* spp.	0	1	0	0	1	0
	*Citrobacter koseri*	0	1	0	0	1	0
	*Enterobacter cloacae*	0	2	1	0	2	1
	*Escherichia coli*	0	16	0	8	8	3
	*Fusobacterium* spp.	0	1	0	1	0	0
	*Hafnia alvei*	1	0	0	1	0	0
	*Klebsiella oxytoca*	0	1	0	1	0	1
	*Klebsiella pneumoniae*	1	6	0	2	5	2
	*Pseudomonas aeruginosa*	1	4	0	1	4	0
	*Salmonella enterica*	0	1	0	1	0	0
	*Serratia marcescens*	0	2	0	2	0	0
**Grampositives**	*Clostridium* spp.	0	0	2	0	0	0
	*Enterococcus faecalis*	1	7	0	8	0	0
	*Enterococcus faecium*	3	5	0	5	3	0
	*Staphylococcus aureus*	0	9	0	3	6	2
	Methicillin-resistant*Staphylococcus aureus*	0	2	0	0	2	0
	Coagulase-negative staphylococci	0	11	0	10	1	0
	*Streptococcus* spp.^1^	0	2	0	0	2	0
	*Streptococcus agalactiae*	0	1	0	0	1	0
	*Streptococcus pneumoniae*	0	5	2	3	2	3
**Yeasts**	*Candida albicans*	0	1	0	1	0	1
**Total**		**7**	**78**	**5**	**47**	**38**	**13**

1 *Streptococcus mitis* and *S. parasanguinis*.

BC, blood culture.

In one case of two closely related microorganisms, the PCR/ESI-MS software misidentified the microorganism (*Klebsiella pneumoniae* as *Citrobacter* spp.), and in a few cases, the identification was only achieved at the genus level (9.6% of the 83 pathogens detected with clinical significance: *Streptococcus* spp., *Citrobacter* spp., *Fusobacterium* spp., *Clostridium* spp. and *Salmonella* spp.). In the case of conventional methods, 3.5% of the 85 microorganisms were also identified at the genus level (*Serratia* spp., *Fusobacterium* spp. and *Bacteroides* spp.).

#### Parameters of analytical performance

The sensitivity, specificity, the PPV and the NPV of the PCR/ESI-MS were 96.7%, 97.7%, 95.2% and 98.5%, respectively, using the BC as the gold standard (**Table 4A**). Given that the molecular method detected a microorganism with clinical value in a negative BC (**Table 5**), these values were 96.8%, 98.5%, 96.8% and 98.5%, respectively when the clinical criterion gold standard was used (**Table 4A**).

**Table 4:** Agreement of unique isolation/detection specimens depending on the gold standard used and the specimen type.

		Blood culture gold standard			Clinical infection criterion		
		Conventional methods			Conventional methods		
		Positive	Negative	Total	Positive	Negative	Total
**A) PCR/ESI-MS in blood culture**	Positive	59	3	**64**	60	2	**64**
	Negative	2^a^	128	**128**	2^b^	128	**128**
	**Total**	**61**	**131**	**192**	**62**	**130**	**192**
**B) PCR/ESI-MS in whole blood**	Positive	28^c^	20	**48**	37	10	**47**
	Negative	36	152	**188**	37	152	**189**
	**Total**	**64**	**172**	**236**	**74**	**162**	**236**

**A)** Blood culture gold standard: 96.7% sensitivity, 97.71% specificity, 95.2% PPV, 98.5% NPV. Clinical infection criterion: 96.8% sensitivity, 98.5% specificity, 96.8% PPV, 98.5% NPV.

a In one specimen an *Enterococcus faecium* was isolated by blood culture whereas PCR/ESI-MS detected a coagulase-negative staphylococci.

b In another specimen a *Klebsiella pneumoniae* was isolated by blood culture and it was identified as *Citrobacter* spp. by PCR/ESI-MS.

**B)** Blood culture gold standard: 43.8% sensitivity, 88.4% specificity, 58.3% PPV, 80.9% NPV. Clinical infection criterion: 50.0% sensitivity, 93.8% specificity, 78.7% PPV, 80.4% NPV.

c The PCR/ESI-MS detected a coagulase-negative staphylococci while a *Klebsiella pneumoniae* was isolated by blood culture. In 10 specimens with a negative paired blood culture (two of them from the same patient and sepsis episode) the PCR/ESI-MS detected clinically significant microorganisms.

**Table 5:** Discrepancies found between the PCR/ESI-MS in blood cultures and conventional methods.

	PCR/ESI-MS results	Conventional methods	Comments
**Clinical evidence supporting** **PCR/ESI-MS results**	CoNS^1^, *Enterobacter cloacae*	*Staphylococcus epidermidis*	Bronchial aspirate culture positive for *E. cloacae* 3 days later. Respiratory superinfection.
	*Streptococcus pneumoniae*	Negative	Pneumonia antecedents. Previous splenectomy. PCR/ESI-MS detected *S. pneumoniae* in the paired whole blood specimen.
	*Bacteroides fragilis, Clostridium* spp.	*Bacteroides* spp.	Abdominal infection. Possible presence of different anaerobic microorganisms.
	*Streptococcus* spp., *Streptococcus* *pneumoniae*, CoNS	*Streptococcus mitis, Staphylococcus epidermidis*	Pneumonia.
	*Fusobacterium* spp.,*Clostridium* spp.	*Fusobacterium* spp.	Soft tissue infection, possible presence of anaerobic microorganisms.
**No clinical evidence supporting** **PCR/ESI-MS results**	*Staphylococcus aureus*	Negative	
	*Staphylococcus aureus*	Negative	Pneumonia.
	*Enterococcus faecalis, Enterobacter* spp.	*Enterococcus faecalis*	Communitarian sepsis. Unknown focus.
	*Klebsiella pneumoniae,* *Citrobacter* spp.	*Klebsiella pneumoniae*	Communitarian sepsis. Unknown focus. Possible misidentification.
**Misidentification**	*Citrobacter* spp.	*Klebsiella pneumoniae*	
**Skin or ambient contaminant**	CoNS	*Enterococcus faecium*	
	*Serratia marcescens, Bacillus* spp.	*Serratia* spp.	

1 *Staphylococcus epidermidis* and other coagulase-negative species.

### 2. Microbial Identification from Whole Blood Specimens

Given that the PCR/ESI-MS technology demonstrated a good performance on BC aliquots, we proceeded with the evaluation in the WB specimens obtained from the same patients.

#### Agreement between isolated/detected microorganisms

From a total of 110 microorganisms identified by either or both methods, 37 were identified both by BC and the PCR-ESI/MS technique, while no identification was achieved in 152 specimens by either method. Thus the overall agreement between methods was 72.1% (κ = 0.316) (**Table 1B**)**.** The PCR/ESI-MS identified a total of 25 microorganisms that were not detected by BC (commented in **Table 6**), and the presence of 13 of them was supported by clinical facts. On the contrary, in four cases the presence of those microorganisms could not be supported by clinical evidence, and another eight microorganisms were considered contaminants from the skin flora that were found in the BC due to inadequate antisepsis before venipuncture (i.e. coagulase-negative staphylococci, *Propionibacterium acnes*, etc.). When the results were reanalyzed taking this clinical information into consideration, the agreement between the PCR/ESI-MS and the conventional methods increased to 77.1% (κ = 0.472) (**Table 1B**). A list of the microorganisms identified by either or both methods is depicted in **Table 3**.

**Table 6:** Discrepancies found between the PCR/ESI-MS in whole blood and conventional methods.

	PCR/ESI-MS results	Conventional methods	Comments
**Clinical evidence supporting** **PCR/ESI-MS results**	*Klebsiella pneumoniae,* *Klebsiella oxytoca*	*Klebsiella pneumonia, Enterococcus faecalis*	Hepatocellular carcinoma and biliar obstruction.
	*Enterococcus faecium,* *Candida albicans*	*Enterococcus faecium*	Intravascular catheter-related sepsis in patient with leukemia and neutropenia treated with caspofungin. *C. albicans* confirmed by sequencing.
	*Escherichia coli,* *Enterobacter cloacae*	*Escherichia coli*	Sepsis of abdominal origin.
	*Escherichia coli*	Negative	Urine culture positive for *E. coli.*
	*Streptococcus pneumoniae*	Negative	Pneumonia in a splectomized patient. Confirmed by sequencing.
	*Staphylococcus aureus*	Negative	Pneumonia due to *S. aureus.*
	*Escherichia coli*	Negative	Urine culture positive for *E. coli.*
	*Streptococcus pneumoniae* 1	Negative	Bilateral pneumonia in an immunocompromised patient with a bronchial aspirate culture positive for *S. pneumoniae.*
	*Klebsiella pneumoniae*	Negative	Previous sepsis due to *K. pneumoniae*. Polycystic kidney disease.
	*Staphylococcus aureus*	Negative	Skin origin of sepsis. Culture positive for *S. aureus.*
	*Klebsiella pneumoniae*	Negative	Bilateral pneumonia. Previous urine culture positive for *K. pneumoniae.*
	*Escherichia coli*	Negative	Acute lymphoblastic leukemia. Primary origin of sepsis. Distended abdomen, possible translocation.
**No clinical evidence** **supporting PCR/ESI-MS results**	*Escherichia coli*	Negative	Pneumonia due to MRSA.^2^
	*Staphylococcus aureus*	Negative	Pneumonia due to *S. pneumoniae.*
	*Escherichia coli*	Negative	Chronic respiratory disease.
	*Escherichia coli*	Negative	
**Skin contaminants**	*Staphylococcus warneri*	*Klebsiella pneumoniae*	
	*Pseudomonas aeruginosa, Corynebacterium* spp.	*Pseudomonas aeruginosa*	
	*Staphylococcus haemolyticus*	Negative	
	*Staphylococcus hominis*	Negative	
	*Staphylococcus epidermidis*	Negative	*S. epidermidis* was detected and considered as contaminant in two specimens, one of them confirmed by sequencing.
	*Propionibacterium acnes*	Negative	*P. acnes* was detected and considered as contaminant in two specimens.

1 *Streptococcus pneumoniae* was detected in two whole blood specimens with negative blood culture from the same patient during the same sepsis episode.

2 Methicillin-resistant *Staphylococcus aureus*.

#### Parameters of analytical performance

Polymicrobial infections were detected by either or both methods in 11 (14.7%) out of the 75 cases with a positive paired BC (**Table 2**). As described above for BC samples, only those specimens with a single pathogen were considered for analysis. When the BC was regarded as the gold standard, the sensitivity, specificity, the PPV and the NPV were 43.8%, 88.4%, 58.3% and 80.9%, respectively. However, taking into account the 10 cases with clinical significance detected by the PCR/ESI-MS only (**Table 6**), the values rose to 50%, 93.8%, 78.7% and 80.4% when the clinical infection criterion was used (**Table 4B**).

Variables such as the time to positivity of the BC, total DNA concentration and leukocyte count were compared between those specimens with a paired positive BC that had a positive detection and those with no detection by PCR/ESI-MS and no statistically significant differences were found (data not shown).

### 3. Detection of Antibiotic Resistances

Among all *Staphylococcus aureus* isolates (N = 11), two were reported to be methicillin-resistant *Staphylococcus aureus* (MRSA) by conventional methods. Both of them were correctly identified by the PCR/ESI-MS technology both in WB and paired BC specimens. Additionally, the presence of the *mecA* gene was reported by the molecular method in two other strains (one from WB and one from BC specimens) that were methicillin-susceptible *Staphylococcus aureus* (MSSA) according to the reference methods.

The other resistance-related genes (*vanA*, *vanB* or *kpc*) were not detected in any of the isolates included in this study by any method.

## Discussion

Sepsis is a major cause of morbidity and mortality in hospitals around the world [21]. Since the rapid administration of an effective antimicrobial therapy is decisive for the best outcome of the patient, a prompt identification of the causal agent is highly desirable to readdress the initial empirical treatment if necessary [3]. This is the first study that clinically evaluated a new technology based on PCR and mass spectrometry for the detection of pathogens directly from WB.

Since sepsis may be caused by a wide diversity of pathogens, mainly bacteria and fungi, the identification of all of them may be challenging for diagnostic assays. Given the low concentration of bacteria/yeast in WB, the ability of the PCR/ESI-MS technology to detect a variety of sepsis-related pathogens was best assessed in BC. Being based on several broad-range PCR reactions, the investigational assay used for the identification of pathogens from BC showed a very good overall agreement (94.2%) with the conventional microbiological methods, with 21 different species identified. These results are in agreement with previous data [22]; 93 (90.3%) of the 103 microorganisms identified by conventional methods were also detected by PCR/ESI-MS (45 different species identified).

Recently, another mass spectrometry-based technology has been adapted to microbiological diagnosis; in this case, a Matrix-Assisted Laser Desorption/Ionization (MALDI-TOF) approach is used to obtain the protein spectrum of microbial pathogens from BC [23]–[25]. E. J. Kaleta *et al.* [26] compared the PCR/ESI-MS technology with the MALDI-TOF approach in BC specimens and a highly accurate identification at species level was achieved with both methodologies (95.2% and 94.3%, respectively). The main advantage of the MALDI-TOF approach is that the identification of pathogens can be achieved within minutes, but a previously grown culture is required, whereas the identification can be directly obtained from uncultured WB in 6–8 hours with the PCR/ESI-MS technology.

As opposed to working with BC aliquots, starting from the patient’s blood would provide microbiologists with the possibility of anticipating the diagnosis up to 40 hours. However, the concentration of bacteria in WB may be as low as 1–10 CFU/mL [27]. Although the PCR/ESI-MS technology was able to detect very low levels of bacteria (1–5 genomes/well) in some cases, its performance was more limited in this specimen type than in BC, as expected. One inherent limitation in comparing molecular methods to culture is that current molecular methods use a smaller volume of blood than BC. This is because an excess of human DNA may hamper the detection of minor bacterial DNA or even inhibit the PCR reaction. Methods to overcome these problems and test larger blood volumes are being developed by the manufacturer as well as other groups [28]–[30]. Even so, our results show that using the PCR/ESI-MS technology, a microbiological diagnosis of sepsis could be achieved directly from the patient’s blood with 50% sensitivity and 93.8% specificity when compared with conventional methods.

Other molecular methods for the diagnosis of sepsis directly from WB have been clinically evaluated. The SeptiFast test is based on a multiplex real-time PCR targeting the 25 most frequent pathogens involved in bloodstream infections. However, the results obtained with this assay are not very consistent across different studies, with sensitivity ranging between 61 and 90.9%, and specificity between 70 and 100% [5]–[10]. The overall agreement between microorganisms isolated by BC and identified with the PCR/ESI-MS in WB in our study was 77.5%, which is comparable to results published using the SeptiFast (69 to 77.8%) [5], [31], [32]. However, microorganisms not included in the mentioned assay were identified in seven cases in our study (*Salmonella enterica, Bacteroides* spp., *Citrobacter koseri*, *Fusobacterium* spp., *Hafnia alvei*, and two cases of *Clostridium* spp.). The VYOO® test (SIRS-Lab, Jena, Germany) is based on a multiplex PCR followed by microarray detection that includes 34 bacterial pathogens, seven fungi and five antibiotic resistance genes, and showed a 46.2% concordance with conventional methods [29]. The SepsiTest (Molzym, Bremen, Germany) uses a universal 16S rRNA PCR followed by sequencing and a concordance of 86% with BC was reported for this assay [30].

While the PCR/ESI-MS offers a quantitative result and it detected from 1 to 399 genomes per PCR well, a lower detection limit is set for the final interpretation of results (3 genomes/well for most pathogens and at 5 genomes/well for the *Candida* spp.). However, this detection limit was risen up to 10 genomes/well by the manufacturer for the coagulase-negative staphylococci (CoNS) and other pathogens likely to be contaminants from the skin flora. While this strategy was designed to improve the specificity of the assay for the diagnosis of sepsis, it could also lead to a low percentage of false-negative results, given that the amount of genomes/well observed for microorganisms with clinical significance and those considered contaminants cannot be easily distinguished. In fact, in nine WB specimens with a positive paired BC the PCR/ESI-MS achieved the correct identifications but they were not reported as they were under the mentioned threshold, being three of them CoNS. On the other hand, eight cases corresponding to skin contaminants were above the limit of detection and, thus, reported as positive. In fact, the detection of such microorganisms is also an issue to be considered when interpreting the results of BC; in our center, in up to 5% of all positive blood cultures a skin contaminant is isolated due to poor aseptic practices at the time of inoculation. Those patients in which a skin contaminant had been identified by BC were excluded from this study.

Several factors may limit the interpretation of the results obtained in our study. Firstly, 42 patients were sampled at several time-points during the same sepsis episode; serial blood cultures are often obtained in the clinical practice for patients that are not evolving favorably. Given that the PCR/ESI-MS results are quantitative, future studies should explore the value of this technique for monitoring antibiotic efficacy and predict clinical outcome of patients with sepsis. Secondly, in some cases the samples were drawn when patients were already under antibiotic treatment. The latter could lead to positive findings by PCR/ESI-MS in sepsis episodes with a negative BC. In the absence of a highly sensitive gold standard, reviewing clinical facts as well as other positive cultures is necessary in these cases. In this study, the clinical relevance of 12 out of 25 such cases was clinically supported by additional positive cultures or by the nature of the underlying infection, and 9 of those 12 patients were on antibiotic treatment. This data supports the fact that molecular technologies may be useful in those cases where the value of traditional culture is limited; the identification of the etiological pathogen in treated patients could have a clinical impact in patient outcome through the adjustment of the initially administered antimicrobial therapy. It also has to be taken into account that molecular methods are able to detect the DNA either from living or dead bacteria, as well as DNA released to the bloodstream by translocation [33], while blood culture and identification by the Vitek-2 system only detects viable microorganisms. Some of the PCR/ESI-MS findings could not be supported by clinical facts and, consequently, the results obtained should always be reviewed and interpreted by a clinical microbiologist considering all the available clinical data.

Despite molecular methods are more expensive than conventional ones, the overall benefits for the patient have to be considered. A rapid identification of the pathogen may lead to the optimization of the administered therapy and, thus, to a prompter recovery of the patient and a shorter stay at the ICU department. Cost/benefit studies regarding the use of molecular assays in combination with conventional methods have been performed using the SeptiFast assay and significant economic savings were reported due to the shortening of the ICU stay and a more rational use of antibiotics [34], [35].

In conclusion, the PCR/ESI-MS technology could be a useful tool to achieve a rapid diagnosis in patients with clinical suspicion of sepsis. Our results show that a significant proportion of patients would beneficiate from an early diagnosis, and its use in combination with traditional methods could increase the number of microbiologically confirmed sepsis cases. Although more studies are necessary to asses the real clinical impact of this technology in the detection of pathogens in whole blood, this early identification of the pathogen could affect the antibiotic treatment and, therefore, the patient management and outcome. In addition, given its capability of detecting any pathogen, this technology offers a high versatility for the diagnosis of infectious diseases.
